# Supplementary material for: Potency of an Inactivated Influenza Vaccine against a Challenge with A/Swine/Missouri/A01727926/2015 (H4N6) in Mice for Pandemic Preparedness
Source: Vaccines (Basel). 2020 Dec 16;8(4):768. doi: 10.3390/vaccines8040768 (PMC7765658; doi:10.3390/vaccines8040768)
Supplement: Supplementary file 1 [file vaccines-08-00768-s001.pdf]

Supplementary Materials:

Table S1. Growth of H4 influenza viruses in embryonated chicken eggs.

Viruses	Group	HA titer of allantoic fluid
A/duck/Czeckoslovakia/1956 (N4N6)	Eurasian	32
A/budgerigar/Hokkaido/1/1977 (H4N6)	Eurasian	128
A/duck/Hokkaido/491003/2014 (H4N2)	Eurasian 1	256
A/duck/Mongolia/769/2015 (H4N6)	Eurasian 2	128
A/duck/Hokkaido/138/2007 (H4N6)	Eurasian 3	128
A/swan/Hokkaido/481102/2017 (H4N6)	Eurasian 4	512
A/mallard/Alberta/223/1979 (H4N2)	North American 1	256
A/swine/Missouri/A01727926/2015 (H4N6)	North American 2	256

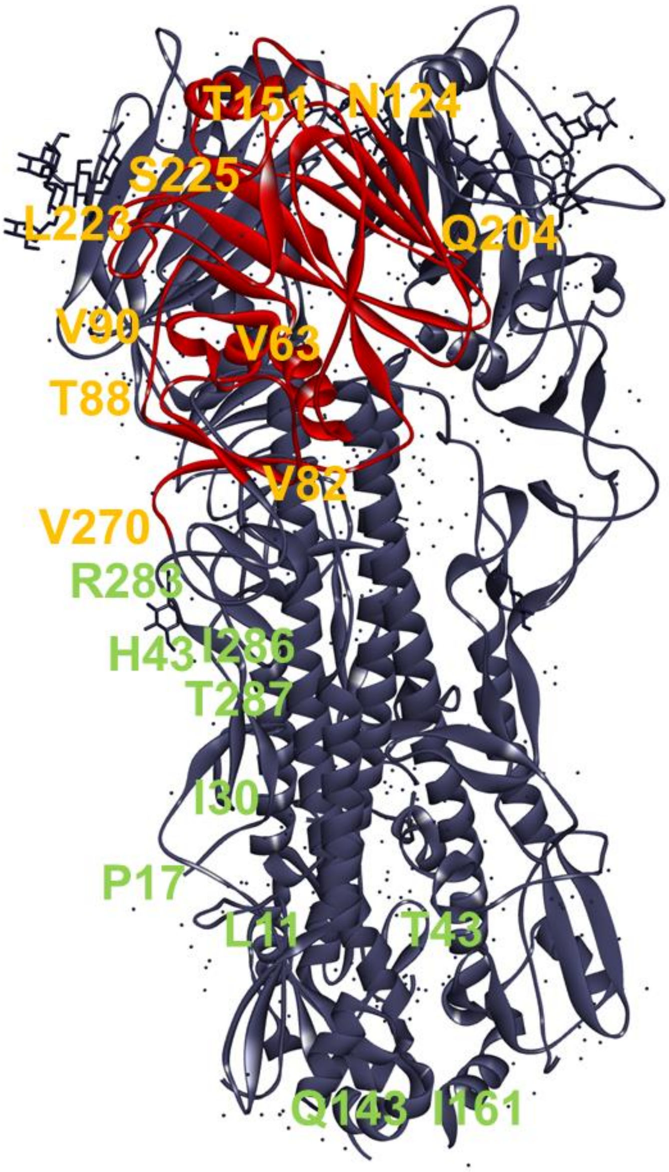

Figure S1. Three-D structure of MO/15 HA trimer and the positions of amino acid substitutions between MO/15 and Swan/Hok. Red: HA1 head domain, Yellow: Different residues at the head domain between MO/15 and Swan/Hok, Green: Different residues at the stalk domain between MO/15 and Swan/Hok.
